# Single Nucleotide Polymorphism in SMAD7 and CHI3L1 and Colorectal Cancer Risk

**DOI:** 10.1155/2018/9853192

**Published:** 2018-10-25

**Authors:** Amal Ahmed Abd El-Fattah, Nermin Abdel Hamid Sadik, Olfat Gamil Shaker, Amal Mohamed Kamal

**Affiliations:** ^1^Biochemistry Department, Faculty of Pharmacy, Cairo University, Kasr El-Einy Street, Cairo, Egypt; ^2^Medical Biochemistry and Molecular Biology Department, Faculty of Medicine, Cairo University, Cairo, Egypt

## Abstract

Colorectal cancer (CRC) is one of the leading cancers throughout the world. It represents the third most common cancer and the fourth in mortality. Most of CRC are sporadic, arise with no known high-penetrant genetic variation and with no previous family history. The etiology of sporadic CRC is considered to be multifactorial and arises from the interaction of genetic variants of low-penetrant genes and environmental risk factors. The most common well-studied genetic variation is single nucleotide polymorphisms (SNPs). SNP arises as a point mutation. If the frequency of the sequence variation reaches 1% or more in the population, it is referred to as polymorphism, but if it is lower than 1%, the allele is typically considered as a mutation. Lots of SNPs have been associated with CRC development and progression, for example, genes of TGF-*β*1 and CHI3L1 pathways. TGF-*β*1 is a pleiotropic cytokine with a dual role in cancer development and progression. TGF-*β*1 mediates its actions through canonical and noncanonical pathways. The most important negative regulatory protein for TGF-*β*1 activity is termed SMAD7. The production of TGF-*β* can be controlled by another protein called YKL-40. YKL-40 is a glycoprotein with an important role in cancer initiation and metastasis. YKL-40 is encoded by the CHI3L1 gene. The aim of the present review is to give a brief introduction of CRC, SNP, and examples of some SNPs that have been documented to be associated with CRC. We also discuss two important signaling pathways TGF-*β*1 and CHI3L1 that influence the incidence and progression of CRC.

## 1. Colorectal Cancer

**Figure 1:**
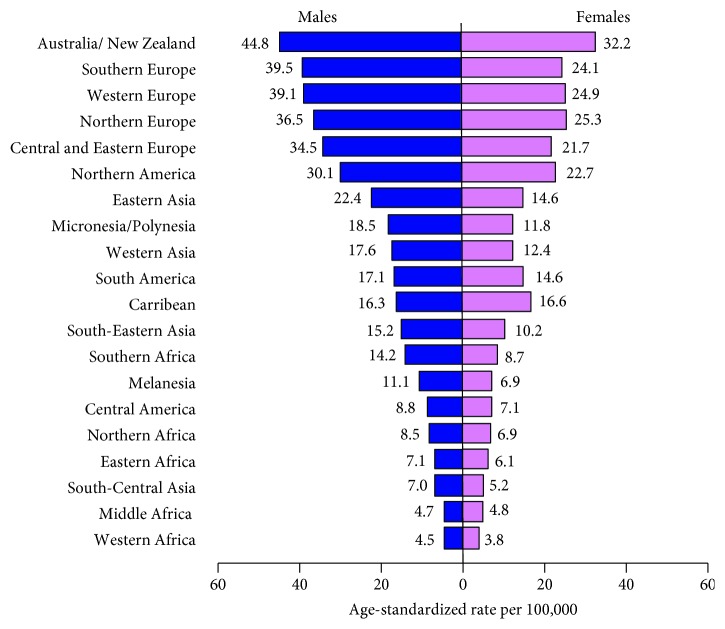
Age-standardized CRC incidence rates by sex and world area, GLOBOCAN 2012.

Colorectal cancer (CRC) has attracted significant attention as it represents the third most common cancer and fourth cancer in mortality in the world after lung, stomach, and liver cancers [1]. Colorectal cancer accounts for approximately 10% of all new cancer cases, affecting one million people every year throughout the world [2]. The highest incidence rates are mainly found in developed countries, whereas the lowest rates are found in developing countries (Figure 1) [3]. From the genetic standpoint, CRC can be divided into three types: sporadic, familial, and hereditary CRC [4] as shown in Table 1.

**Figure 2:**
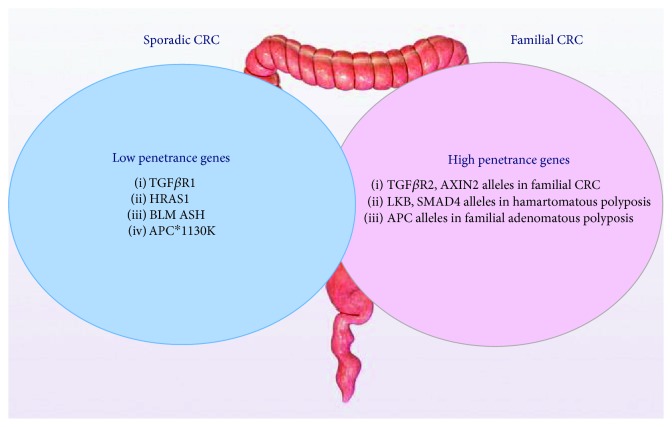
Genetic contribution to CRC.

The etiology of sporadic CRC is considered to be multifactorial and arises from the interaction between allelic variants in low-penetrant genes and environmental risk factors [5, 6]. Penetrance is the frequency with which the characteristics transmitted by a gene appear in individuals possessing it. A highly penetrant gene almost always expresses its phenotypes regardless of other environmental influence, while low-penetrant genes express its phenotype in the presence of other genetic and/or environmental influence [7]. The genetic contribution of high- and low-penetrant genes to CRC is shown in Figure 2. Risk factors for CRC may be nonmodifiable or modifiable [8] as shown in Table 2.

**Figure 3:**
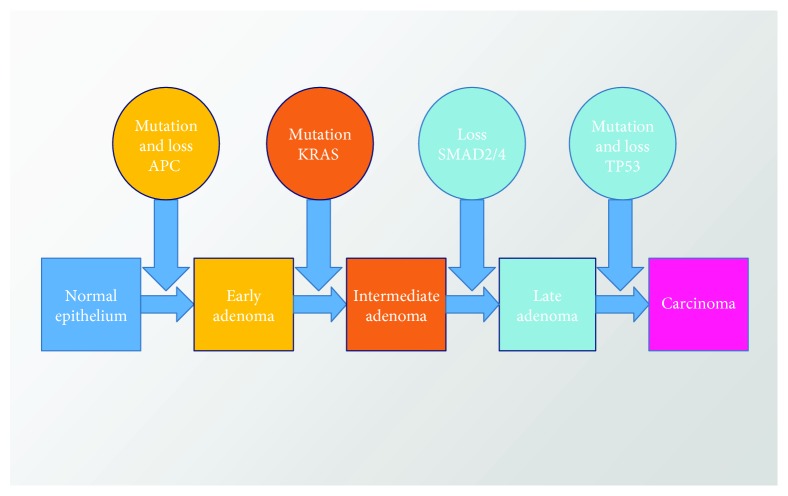
The colorectal adenoma-carcinoma sequence (Vogelstein model). Progression from normal epithelium through adenoma to CRC is characterized by accumulated abnormalities of multiple genes.

Vogelstein model, also known as the adenoma-carcinoma sequence, is a multistep model [19] that describes the progression of CRC carcinogenesis from a benign adenoma to a malignant carcinoma through a series of well-defined histological stages (Figure 3). The main features of the model include a mutational activation of oncogenes and/or the inactivation of tumor suppressor genes. At least four or five genetic alterations must take place for the formation of malignant tumors. The characteristics of the tumor are dependent upon the accumulation of multiple genetic mutations rather than a certain sequence of mutations of these genes.

Dukes' colorectal cancer staging and Tumors/Nodes/Metastases (TNM) are the two classification system that are used for the staging of CRC (Table 3). There has been a gradual move from Dukes' to the TNM classification system as TNM was reported to give a more accurate independent description of the primary tumors and its spread [20].

## 2. Prevention of Colorectal Cancer

Several approaches have been developed to reduce CRC incidence and mortality. Prevention includes primary and secondary strategies. Primary strategy includes dietary changes, increasing physical activity, and the use of nonsteroidal anti-inflammatory drugs (NSAIDs), while the secondary strategy is based on screening tests (Table 4).

Interestingly, dietary factors are responsible for 70% to 90% of CRC. The relatively low CRC rates in the Mediterranean area compared with most Western countries are mostly because the traditional Mediterranean diet is characterized by high consumption of foods of plant origin, relatively low consumption of red meat, and high consumption of olive oil [32]. Therefore, diet modification could potentially help to reduce the incidence of CRC [33, 34]. Examples of some dietary components that lower CRC risk are shown in Table 5.

Early diagnosis of CRC is important to improve outcomes. Fecal occult blood testing (FOBT) or fecal immunochemical test (FIT) is routinely used prior to colonoscopy, and only patients with a positive test result are referred to a specialist. Although these assays are useful screening tools, patient compliance with these stool-based assays tends to be low. Serum-based assays for the early detection of CRC are highly attractive, as they could be integrated into any regular health checkup without the need for additional stool sampling, thereby increasing acceptance among patients [29].

## 3. Gene Polymorphism

Polymorphism is the occurrence of two or more clearly different morphs or forms of a species in the population. Poly means many; morph means form [48]. The colored flowers of mustard, butterflies, and human ABO blood group system are obvious examples of polymorphisms [49, 50].

Genetic polymorphisms are different forms of the DNA sequence, which may or may not affect biological function depending on its exact nature. Polymorphism arises as a result of mutation. If the frequency of a specific sequence variant reaches 1% or more in the population, it is referred to as polymorphism, and if it is lower than 1%, the allele is typically regarded as mutation [51]. Molecular polymorphism, first demonstrated in *Drosophila pseudoobscura*, stimulated molecular studies of many other organisms and led to vigorous theoretical debate about the significance of the observed polymorphisms [52, 53].

Single nucleotide polymorphism (SNP) is a variation in a single nucleotide that occurs at a specific position in the genome. Single nucleotide polymorphisms are the most abundant type of genetic variation in the human genome, accounting for more than 90% of all differences between individuals [54]. Single nucleotide may be changed (substitution), removed (deletion), or added (insertion) to a polynucleotide sequence [54].

Single nucleotide polymorphisms are also thought to be the keys in realizing the concept of personalized medicine as it can affect how humans develop diseases and respond to pathogens, chemicals, drugs, vaccines, and other agents. Single nucleotide polymorphisms underlie the differences in the susceptibility to a wide range of human diseases, for example, a single base mutation in the apolipoprotein E gene is associated with a higher risk for Alzheimer's disease. The severity of illness and the way the body responds to treatments are also manifestations of genetic variations [55, 56].

According to their location in the genome, SNPs are classified into cSNP in the coding region (exons), rSNP in the regulatory region, and iSNP located in the intronic region [54].

**Figure 4:**
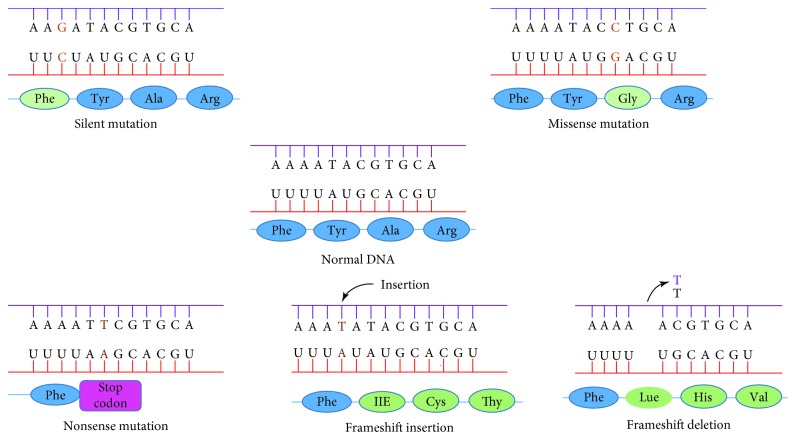
Genetic polymorphism in the coding region (http://academic.pgcc.edu/).

Polymorphisms in the coding region are either synonymous or nonsynonymous (Figure 4). Synonymous polymorphisms do not result in a change of amino acid in the protein but still can affect its function in other ways. Silent mutation in the multidrug resistance gene 1, which codes for a cellular membrane pump that expels drugs from the cell, is an example of synonymous polymorphism. It can slow down translation and allow unusual folding of the peptide chain, causing the mutant pump to be less functional [57, 58].

Nonsynonymous polymorphisms, on the other hand, can change the amino acid sequence of the protein and subclassified into missense and nonsense. Missense polymorphism results in different amino acids such as single base change G > T in LMNA gene that results in the replacement of the arginine by the leucine at the protein level, which manifests progeria syndrome [59]. Nonsense polymorphism results in a premature stop codon and usually nonfunctional protein product such as that manifested in cystic fibrosis caused by mutation in the cystic fibrosis transmembrane conductance regulator gene [60].

Promoter polymorphism can cause variations in gene expression as it affects the DNA binding site and alters the affinity of the regulatory protein while intronic region polymorphism may affect gene splicing and messenger RNA degradation [61, 62].

Genotyping technologies typically involve the generation of allele-specific products for SNPs of interest followed by their detection for genotype determination. All current genotyping technologies with only a few exceptions require the polymerase chain reaction (PCR) amplification step. In most technologies, PCR amplification of a desired SNP-containing region is performed initially to introduce specificity and increase the number of molecules for detection following allelic discrimination [63]. Enzymatic cleavage, primer extension, hybridization, and ligation are four popular methods used for allelic discrimination (Table 6).

## 4. Genome-Wide Association Study and Colorectal Cancer

Genome-wide association study (GWAS), also known as whole genome association study, is defined as an examination of many common SNPs in different individuals to see if any SNP is associated with a disease. Genome-wide association study compares the DNA of participants having a disease with similar people without the disease. The ultimate goal is to determine genetic risk factors that can be used to make predictions about who is at risk for a disease and to identify their role in disease development for developing new prevention and treatment strategies [68].

The availability of chip-based microarray technology that assay hundreds and thousands of SNPs made genome-wide association studies easy to be performed (Table 7). Genome-wide association study identifies a specific location, not complete genes. Many SNPs identified in GWAS are near a protein-coding gene or are within genes that were not previously believed to associate with the disease. So, researchers use data from this type of study to pinpoint genes that may contribute to a person's risk of developing a certain disease [69].

Genome-wide association study is built on the expanding knowledge of the relationships among SNPs generated by the international HapMap project. The HapMap project is an international scientific effort to identify common SNPs among people from different ethnic populations. When several SNPs cluster together on a chromosome, they are inherited as a block known as a haplotype. The HapMap describes haplotypes, including their locations in the genome, and how common they are present in different populations throughout the world [70].

Genome-wide association study is an important tool for discovering genetic variants influencing a disease, but it has important limitations, including their potential for false-positive and false-negative results and for biases related to selection of study participants and genotyping errors [71]. The gold standard for validation of any GWAS is replication in an additional independent sample. Replication studies are performed in an independent set of data drawn from the same population as the GWAS, in an attempt to confirm the effect in the GWAS target population. Once an effect is confirmed in the target population, other populations may be sampled to determine if the SNP has an ethnic-specific effect [72].

It has been recognized that SNPs play an important role in conferring risk of CRC. Genome-wide association studies have reported multiple risk loci associated with risk CRC, some of which are involved in the transforming growth factor-*β* (TGF-*β*) signaling pathway [73]. For example, SMAD7 rs4939827 was found to be associated with CRC in two GWASs [74, 75]. The association of SMAD7 rs4939827 with CRC was confirmed by other replication studies [76, 77]. A summary of other SNPs studied as risk factors for CRC is shown in Table 8.

## 5. Transforming Growth Factor-*β* Signaling and Its Regulatory Smad7

Mothers against decapentaplegic homolog 7 (Smad7) is a key inhibitor of TGF-*β* [94, 95]. Smad7 was named after mothers against decapentaplegic (mad), an intermediate of the decapentaplegic signaling pathway in *Drosophila melanogaster* and sma-gene in *Caenorhabditis elegans* that has mutant phenotype similar to that observed for the TGF-*β*-like receptor gene [96]. Regulation of TGF-*β* by Smad7 is crucial to maintain gastrointestinal homeostasis [97]. Smad7 overexpression is commonly found in patients with chronic inflammatory conditions of the colon [98] and may be associated with prognosis in patients with CRC [99]. Loss of Smad/TGF-*β* signaling interrupts the principal role of TGF-*β* as a growth inhibitor, allowing unchecked cellular proliferation [100].

In the early 1980s, Roberts and his colleagues isolated two fractions that could induce growth of normal fibroblasts from murine sarcoma cell extracts and were named TGF*α* and TGF-*β* [101, 102]. Transforming growth factor-*β* is a prototype of a large family of cytokines that includes the TGF-*β*s, activins, inhibins, and bone morphogenetic proteins (BMPs) [103].

In mammals, TGF-*β* has 3 isoforms (TGF-*β*1, TGF-*β*2, and TGF-*β*3), with similar biological properties. The TGF-*β* isoforms are encoded from genes located on different chromosomes. The TGF-*β*1 gene is located in chromosome 19q13.1, while TGF-*β*2 and TGF-*β*3 genes are located in chromosomes 1q4.1 and 14q24.3, respectively [104].

**Figure 5:**
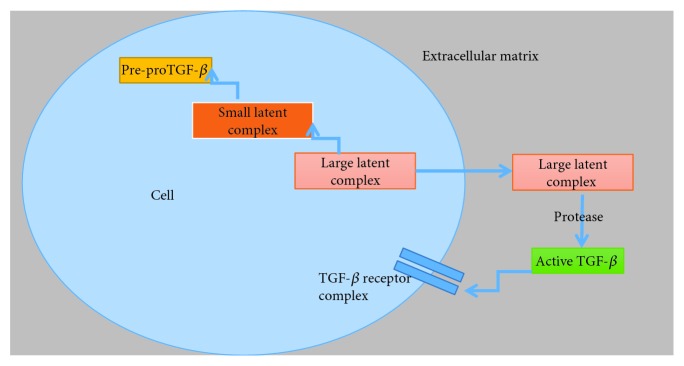
The sequential steps in the synthesis and secretion of active TGF-*β*.

The isoforms of TGF-*β*1, TGF-*β*2, and TGF-*β*3 are encoded as large precursor, which undergo proteolytic digestion by the endopeptidase furin, yielding two products that assemble into dimers. One is latency-associated peptide (LAP), a dimer from the N-terminal region. The other is mature TGF-*β*, a dimer from the C-terminal portion. A common feature of TGF-*β* is that its N-terminal portion (LAP) remains noncovalently associated with the mature TGF-*β* forming a small latent complex [105, 106]. The small latent complex is associated with a large protein termed latent TGF-*β* binding protein (LTBP) via disulfide bonds forming large latent complex for targeted export to the extracellular matrix (ECM) [107, 108]. For TGF-*β* to bind its receptors, the latent complex must be removed so that the receptor-binding site in TGF-*β* is not masked by LAP. Latent TGF-*β* is cleaved by several factors, including proteases, thrombospondin, reactive oxygen species (ROS), and integrins (Figure 5) [109, 110].

Transforming growth factor-*β* is a pleiotropic cytokine that has a dual function in cancer development, where it acts as a tumor suppressor in the early stages and a tumor promoter in the late stages [111]. The main actions of TGF-*β* are summarized in Table 9.

**Figure 6:**
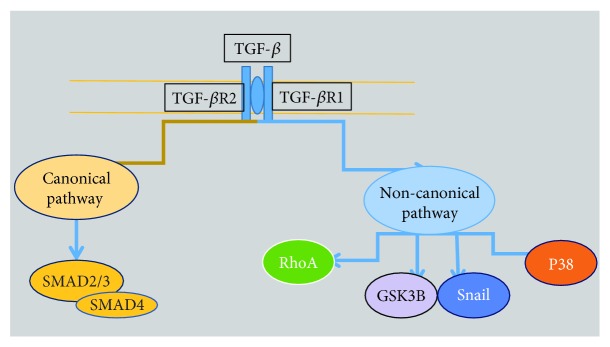
Canonical and noncanonical pathways of TGF-*β*.

The active TGF-*β* binds to transforming growth factor-*β* receptor 2 (TGF-*β*R2), a serine/threonine kinase receptor, leading to the recruitment and phosphorylation of the TGF-*β*R1 (Figure 6). The activated TGF-*β*R1 interacts with and phosphorylates a number of proteins, thereby activating multiple downstream signaling pathways in either a Smad-dependent (canonical) or Smad-independent (noncanonical) signaling pathway (Figure 6) [96].

In the canonical pathway, TGF-*β*R1 propagates the signal through a family of intracellular signal mediators known as Smads. To date, eight mammalian Smad proteins have been characterized and are grouped into three functional classes: receptor-activated Smads (R-Smads) including Smad1, Smad2, Smad3, Smad5, and Smad8, common mediator Smad (Smad4), and inhibitory Smads (I-Smads) including Smad6 and Smad7. Receptor-activated Smads are retained in the cytoplasm by binding to SARA (Smad anchor for receptor activation). Receptor-activated Smads are released from SARA when they are phosphorylated by the activated TGF-*β*R1 [130, 131].

Once R-Smads (Smad2/3) are activated through phosphorylation by TGF-*β*R1, they form an oligomeric complex with Smad4 and translocate into the nucleus, where it modulates the transcription of specific genes. Ability of Smads to target a particular gene and the decision to activate or repress gene transcription are determined by many cofactors that affect the Smad complex [130].

In the noncanonical pathway, TGF-*β* activates other non-Smad signaling pathways (Table 10). Some of these pathways can regulate Smad activation, but others might induce responses unrelated to Smad [132].

Transforming growth factor-*β* is strongly implicated in cancer as genetic alterations of some common components of TGF-*β* pathway (Table 11) that have been identified in human tumors [141].

## 6. Inhibitory Smad (I-Smad, Smad7)

**Figure 7:**
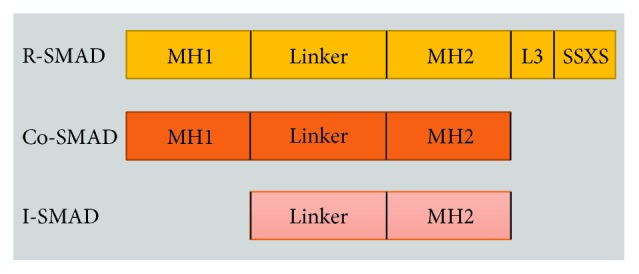
Gene constructions of SMADs.

Mothers against decapentaplegic homolog 7 (Smad7) belongs to the third type of Smads, the I-Smads that also include Smad6. The structure of the Smads is characterized by two conserved regions known as the amino terminal (N-terminal) Mad homology domain-1 (MH1) and C-terminal Mad homology domain-2 (MH2), which are joined by a short poorly conserved linker region. The MH1 domain is highly conserved among the R-Smads and the Co-Smad, whereas the I-Smads lack a MH1. The MH2 domain is conserved among all of the Smad proteins but I-Smads lack SXSS motif, which is needed for phosphorylation following TGF-*β*R1 activation (Figure 7). Thus, I-Smads are not phosphorylated upon binding of TGF-*β* to its receptors. The L3 loop in the MH2 domain of the R-Smads is a specific binding site for the TGF-*β*R1 [95, 156].

**Figure 8:**
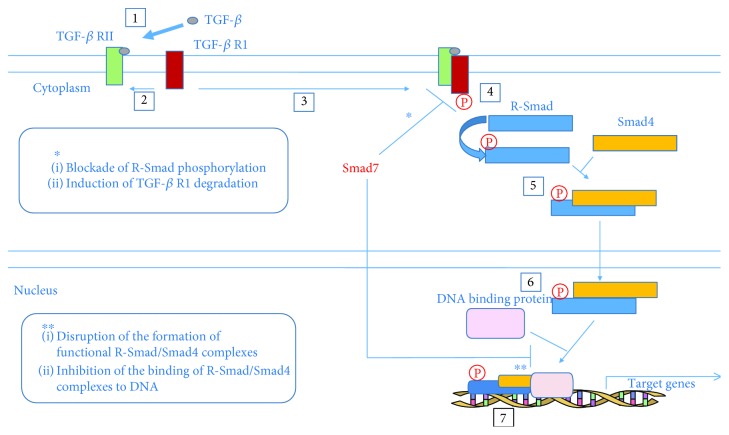
Smad7 antagonizes TGF-*β* signaling in the cytoplasm and the nucleus, respectively [160].

Smad7 antagonizes TGF-*β* signaling through multiple mechanisms, both in the cytoplasm and the nucleus (Figure 8). Smad7 antagonizes TGF-*β* in the cytoplasm through the formation of a stable complex with TGF-*β*R1, leading to inhibition of R-Smad phosphorylation. Smad7 can recruit E3 ubiquitin ligases that induce the degradation of activated TGF-*β*R1 complexes [156, 157]. Also, Smad7 forms a heteromeric complex with R-Smads through the MH2 domain and hence interferes with R-Smad (Smad2/3)-Smad4 oligomerization in a competitive manner. Additionally, Smad7 can bind to DNA disrupting the formation of functional Smad-DNA complexes [158, 159].

Inhibitory Smads can mediate the cross talking of TGF-*β* with other signaling pathways. Various extracellular stimuli such as interferon-*γ* (IFN-*γ*) can induce Smad7 expression to exert opposite effects on diverse cellular functions modulated by TGF-*β* [161]. In addition, Smad7 was found to be a key regulator of Wnt/*β*-catenin pathway that is responsible for the TGF-*β*-induced apoptosis and survival in various cell types [162].

There is a controversy regarding the role of Smad7 in tumor development depending on the type of the tumor. High Smad7 expression was reported to be correlated with the clinical prognosis of patients with colorectal, pancreatic, liver, and prostate cancer. In contrast, a protective role of high Smad7 expression was reported in other tumors [163]. Boulay et al. [164] found that CRC patients with deletion of Smad7 had a favorable clinical outcome compared with patients with Smad7 expression. Additionally, Smad7 was found to act as a scaffold protein to facilitate TGF-*β*-induced activation of p38 and subsequent apoptosis in prostate cancer cells [162].

Even in the same tumor, the function of Smad7 can switch from tumor suppressive to tumor promoting depending on the tumor stage (i.e., early versus advanced). These apparently contradictory functions are in harmony with the opposite roles of TGF-*β* signaling pathway in the early versus advanced tumor stages and the interaction of Smad7 with a vast array of functionally heterogeneous molecules that may be differently expressed during the carcinogenic process [160].

The overexpression of Smad7 in CRC cell was reported to enhance cell growth and inhibit apoptosis through a mechanism dependent on suppression of TGF-*β* signaling [100]. In addition, Smad7-deficient CRC cells were reported to enhance the accumulation of CRC cells in S phase of cell cycle and cell death through a pathway independent on TGF-*β* [165]. Genetic variants in SMAD7 gene have been extensively studied in CRC patients (Table 12).

## 7. Chitinase 3 Like 1/YKL-40

YKL-40 is a mammalian member of the chitinase protein family. YKL-40 is a 40 kDa heparin- and chitin-binding glycoprotein. The human protein was named YKL-40 based on its three N-terminal amino acids tyrosine (Y), lysine (K), and leucine (L) and its 40 kDa molecular mass [178]. This protein has several names, YKL-40 [178], human cartilage glycoprotein-39 (HC-gp39) [179], 38 kDa heparin-binding glycoprotein (Gp38k) [180], chondrex [181], and 40 kDa mammary gland protein (MGP-40) [182].

In a search of new bone proteins, the glycoprotein YKL-40 was identified in 1989 to be secreted in vitro by the human osteosarcoma cell line MG63. The protein was later found to be secreted by differentiated smooth muscle cells, macrophages, human synovial cells, and nonlactating mammary gland [178, 181, 182]. In 1997, the chitinase 3 like 1 (CHI3L1) gene encoding for YKL-40 was isolated. It is assigned to chromosome 1q31-q32 and consists of 10 exons and spans about 8 kilobases of genomic DNA [178, 183].

Based on amino acid sequence, it was found that YKL-40 belongs to the glycosyl hydrolase family 18 that hydrolyses the glycosidic bond between two or more carbohydrates or between a carbohydrate and a noncarbohydrate moiety. Based on sequence similarity, there are more than 100 different families of glycosyl hydrolases [184–186].

Chitin, a polymer of N-acetyl glucosamine, is the second most abundant polysaccharide in nature, following cellulose. It is found in the walls of fungi, the exoskeleton of crabs, shrimp and insects, and the micro filarial sheath of parasitic nematodes [187]. Chitin accumulation is regulated by the balance of chitin synthase-mediated biosynthesis and degradation by chitinases. Although YKL-40 contains highly conserved chitin-binding domains, it functionally lacks chitinase activity due to the mutation of catalytic glutamic acid into leucine [183].

Several types of solid tumors can express YKL-40 such as osteosarcoma [178], CRC [188], thyroid carcinoma [189], breast [190], ovarian [191], lung [192], pancreatic cancer [193], glioblastoma [194–196], and cholangiocarcinoma [197].

**Figure 9:**
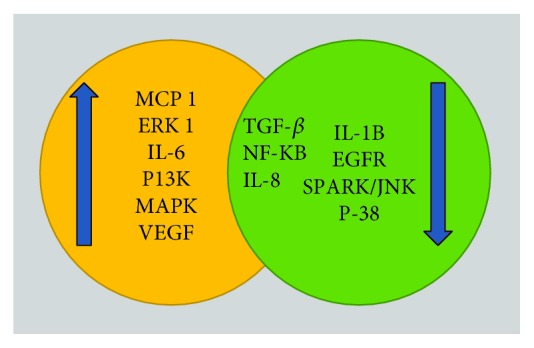
Several synergistic and antagonistic factors modulate the regulatory functions of YKL-40. EGFR: epidermal growth factor receptor; SAPK: stress-activated protein kinases; MCP-1: monocyte chemoattractant protein-1.

There are several synergistic and antagonistic factors that modulate the regulatory functions of YKL-40 (Figure 9) in both normal and pathological conditions [198].

## 8. CHI3L1/YKL-40 Targets and Actions

**Figure 10:**
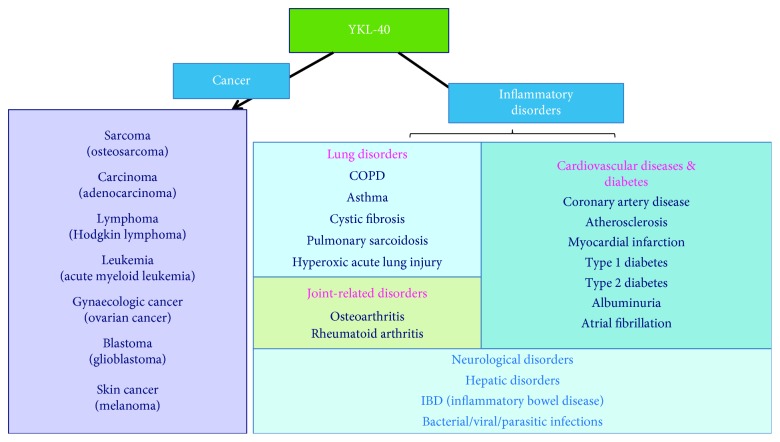
YKL-40 regulates the pathogenesis of cancer and inflammatory disorders [198].

Although the biological function of YKL-40 is not fully understood, the pattern of its expression suggests function in remodeling or degradation of ECM. The diverse roles of YKL-40 in cell proliferation, differentiation, survival, inflammation, and tissue remodeling have been suggested [199]. Aberrant expression of YKL-40 is associated with the pathogenesis of an array of human diseases (Figure 10).

Elevated serum YKL-40 levels were reported to be associated with a wide range of inflammatory diseases (Table 13). More than 75% of patients with streptococcus pneumoniae bacteremia had elevated serum levels of YKL-40 compared with age-matched healthy subjects. Treatment of these patients with antibiotics resulted in reaching serum YKL-40 normal level within few days in most patients before the serum C-reactive protein (CRP) reach the normal level [200].

**Figure 11:**
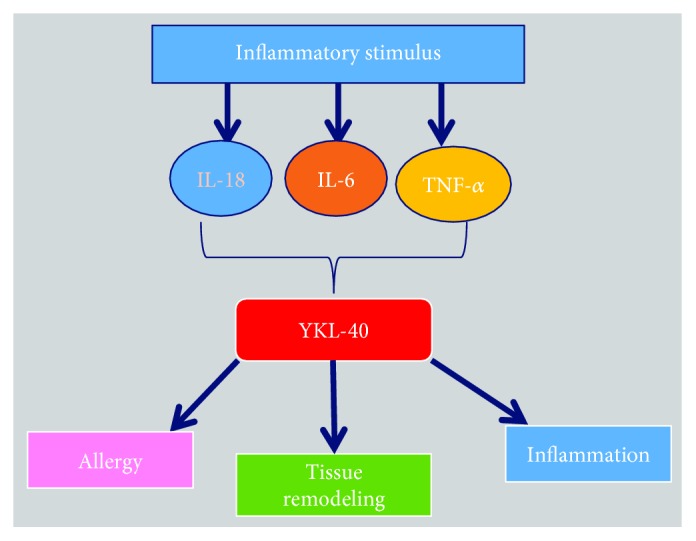
Role of inflammatory cytokines in YKL-40-mediated allergy and inflammation.

Biologically, YKL-40 was found to activate a wide range of inflammatory responses. An inflammatory stimulus can trigger the secretion of a variety of cytokines that in turn may regulate YKL-40 (Figure 11). Increased YKL-40 was reported to regulate chronic inflammatory responses like asthma, chronic obstructive pulmonary disease (COPD), cardiovascular disease (CVD), and arthritis. Inhibition of YKL-40 by utilizing anti-CHI3L1 antibody may be a useful therapeutic strategy to control/reduce the effect of inflammatory diseases [198].

Over the past three decades, a considerable attention has been focused on the potential role of YKL-40 in the development of a variety of human cancers. Serum levels of YKL-40 (Table 14) were independent of serum carcinoembryonic antigen (CEA) in CRC [188], serum cancer antigen 125 (CA-125) in ovarian cancer [191], serum human epidermal growth factor receptor 2 (HER-2) in metastatic breast cancer [190], serum lactate dehydrogenase (LDH) in small cell lung cancer [192], and serum prostate-specific antigen (PSA) in metastatic prostate cancer [208]. Therefore, it may be of value to include serum YKL-40 as a biomarker for screening of cancer together with a panel of other tumor markers as it can reflect other aspects of tumor growth and metastasis than the routine tumor markers [201].

**Figure 12:**
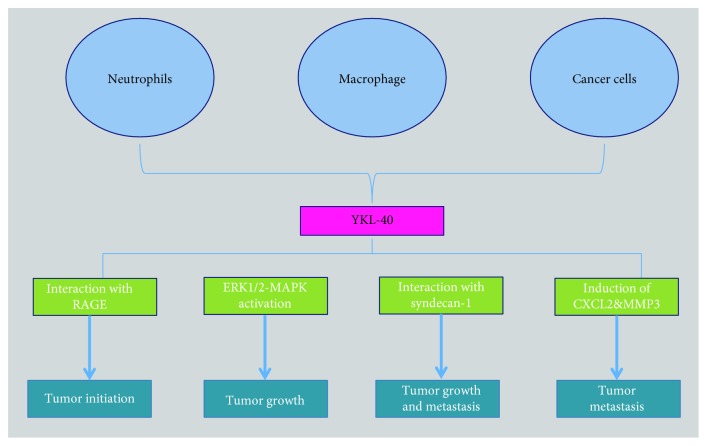
YKL-40 supports tumor progression.

Macrophages and neutrophils in tumor microenvironment or tumor cells were found to secrete YKL-40 into extracellular space, which can enhance tumor initiation, proliferation, angiogenesis, and metastasis (Figure 12).

The ability of YKL-40 to induce cytokine secretion, proliferation, and migration of target cells suggests the existence of their receptors on the cell surface. However, receptors interacting with YKL-40 are incompletely characterized, and only limited information is available about YKL-40-induced signaling pathways. There are evidences to strengthen a hypothesis that a cross talk between adjacent membrane-anchored receptors plays a key role in transmitting “outside-in” signaling to the cells, leading to a diverse array of intracellular signaling [213, 214].

**Figure 13:**
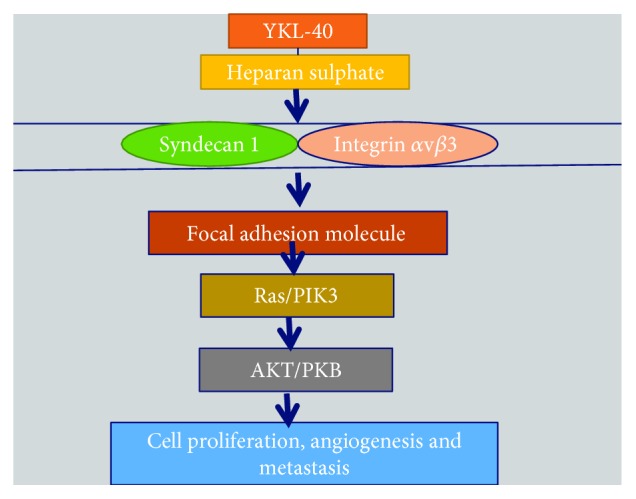
Involvement of YKL-40 in pathways pertaining to cell proliferation, survival, differentiation, and tumorigenesis.

YKL-40 possesses heparin-binding affinity, which enables it to specifically bind heparan sulfate (HS) fragments [215]. Syndecans are transmembrane molecules with cytoplasmic domains that can interact with a number of regulators [216]. Syndecan-1 is the major source of cell surface HS. There is compelling evidence demonstrating that syndecan-1 can act as a matrix coreceptor with adjacent membrane-bound receptors such as integrins to mediate cell adhesion and/or spreading [217]. It was found that YKL-40 could induce the coupling of syndecan-1 and *α*v*β*3 integrin (Figure 13), resulting in phosphorylation of focal adhesion kinase (FAK) and activation of downstream ERK1/2 signaling pathway, which enhance vascular endothelial growth factor (VEGF) expression in tumor cells, angiogenesis, and tumor growth [214]. Additionally, ERK1/2 and JNK signaling pathways were reported to upregulate proinflammatory mediators such as C-chemokine ligand 2 (CCL2), chemokine CX motif ligand 2 (CXCL2), and MMP-9; all of which contribute to tumor growth and metastasis [218].

Another VEGF-independent pathway was reported to mediate angiogenic activity of YKL-40, as an anti-VEGF neutralizing antibody failed to impede YKL-40-induced migration [219]. Therefore, targeting both YKL-40 and VEGF could be an efficient course of therapy along with radiotherapy for eventual eradication of deadly diseases.

**Figure 14:**
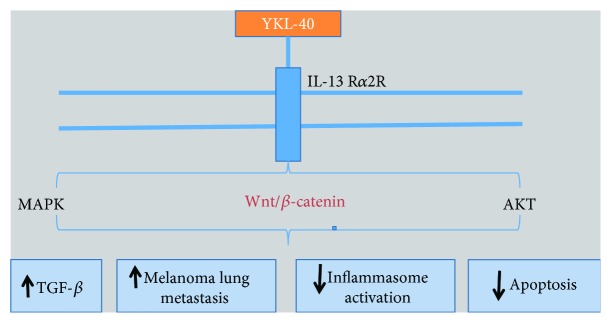
YKL-40 function through IL-13R*α*2-dependent mechanism.

Furthermore, YKL-40 was demonstrated to stimulate TGF-*β*1 production in malignant cells via interleukin-13 receptor *α*2- (IL-13R*α*2-) dependent mechanism (Figure 14). The binding of YKL-40 to IL-13R*α*2 results in the activation of MAPK, AKT, and Wnt/*β*-catenin which play an important role in inhibiting apoptosis and interleukin-1*β* (IL-1*β*) production thereby acting as a potential cancer promoter [220].

Recently, Low et al. [221] showed that YKL-40 can also bind surface receptor for advanced glycation end product (RAGE), which is involved in tumor cell proliferation, migration, and survival through *β*-catenin- and nuclear factor kappa-B- (NF-*κ*B-) associated signaling pathways [221, 222].

Most of the ongoing researches have been carried out on SNP rs4950928 in the promoter region of CHI3L1 gene as it was found to be associated with the serum/plasma YKL-40 levels [223, 224] and diseases such as asthma, bronchial hyperresponsiveness [207], and the severity of hepatitis C virus-induced liver fibrosis [225]. Some of the association studies of CHI3L1 SNPs with different diseases are shown in Table 15.

## Figures and Tables

**Table 1:** Genetic classification of CRC.

Sporadic CRC	Familial CRC	Hereditary CRC
Occurs entirely by chance throughout life without any previous family history	Occurs when there are two or more family members with a history of CRC	When people inherit a high penetrant gene mutation from either of their parents
	No specific inherited gene mutation has been identified to explain the cancer yet.	

~60%–80%	~15%–30%	~5%

**Table 2:** Risk factors of CRC.

*Nonmodifiable*
(i) Age: the incidence of CRC diagnosis increases after the age of 40 and rises sharply after age 50, but there is an increase in the young-onset rate due to the adoption of a Westernized lifestyle and diet [9] (ii) Family history of CRC (especially a first-degree relative diagnosed at age 49 or younger) [10] (iii) Hereditary predisposition (a) Hereditary nonpolyposis colorectal cancer (HNPCC, Lynch syndrome) (b) Familial adenomatous polyposis (FAP) [4, 9] (iv) Inflammatory bowel disease (IBD): chronic inflammation is assumed to underlie the cause of colitis-associated cancer, which is associated with oxidative stress-induced DNA damage resulting in the activation of procarcinogenic genes and silencing of tumor-suppressor pathways [11] (v) Adenomatous polyp: polyps are abnormal growths of the large intestine lining that protrude into the intestinal lumen. Polyps greater than one centimeter in diameter are associated with a greater risk of cancer [12]

*Modifiable*
(i) Diets: Western diet rich in red meat, refined grains, desserts, and low in fiber was reported to be associated with increased CRC risk [10, 13, 14] (ii) Cigarette smoking: carcinogens as aromatic amines, nitrosamines, and polycyclic aromatic hydrocarbons in tobacco smoke produce metabolites that can react with DNA or other macromolecules to form DNA adducts inducing genetic mutations [15] (iii) Obesity: obese women have higher risk of CRC than obese men due to higher abdominal visceral adipose tissue volume [16, 17] (iv) High alcohol consumption (>2 glasses per day): ethanol increases the activation of various procarcinogens present in tobacco smoke, diets, and industrial chemicals to carcinogens through the induction of CYP2E1 [18]

**Table 3:** Staging and survival of CRC.

Dukes' staging	TNM staging	Description	Survival (%)
	Stage 0	Carcinoma in situ	
A	Stage I	No nodal involvement, no metastasis, tumor invades submucosa (T_1_, N_0_, M_0_), tumor invades muscularis (T_2_, N_0_, M_0_)	90–100%
B	Stage II	No nodal involvement, no metastasis, tumor invades subserosa (T_3_, N_0_, M_0_), invade other organ (T_4_, N_0_, M_0_)	75–85%
C	Stage III	Regional lymph nodes involved (any T, N_1_, M_0_)	30–40%
D	Stage IV	Distant metastasis (any T, any N, M_1_)	<5%

**Table 4:** Primary and secondary prevention strategies of CRC.

*Primary*
(i) *Diet*. A diet high in vegetables, fruits, dairy products, olive oil, fish, and whole grains and low in red and processed meats has been shown to lower CRC risk [21–23].
(ii) *Physical Activity*. Physically active individuals have 24% lower risk of CRC development than those who have a sedentary lifestyle. Physical activity promotes the production of interleukin-6 (IL-6) and decreases the expression of inducible nitric oxide synthase (iNOS) and tumor necrosis factor-alpha (TNF-*α*) in plasma and colon, leading to enhanced immunity [24, 25].
(iii) *NSAIDs*. They reduce the risk of CRC by blocking cyclooxygenase (COX) enzymes, so inhibit prostaglandin production, which are known to promote tumor angiogenesis and cell proliferation [26].

*Secondary*
(i) *Fecal Tests*. Fecal occult blood test (FOBT) and fecal immunochemical test (FIT) detect hidden blood in the stool, while fecal DNA test detects DNA in the stool [27–29].
(ii) *Flexible Sigmoidoscopy*. It is performed using an endoscope that allows the examination of the surface up to 60 cm from the anal verge (rectum, sigmoid colon, and part of the descending colon). It is done after colon lavage using enema or administering laxatives without the need of sedation [30].
(iii) *Colonoscopy*. It is performed using an endoscope, which allows an examination of the entire colon surface. It must be done under intravenous sedation and requires being on a low-residue diet, colon lavage using laxatives, and drinking plenty of water the day before the test [31].

**Table 5:** Examples of some dietary components that decrease risk of CRC.

Fiber	(i) A high-fiber diet has a protective effect from CRC as it decreases transit time through the gastrointestinal tract, dilutes colonic contents, and enhances bacterial fermentation. This can increase the production of short-chain fatty acids that interfere with numerous regulators of the cell cycle, proliferation, and apoptosis such as *β*-catenin, p53, and caspase 3 genes [35, 36] (ii) Corn, beans, avocado, brown rice, lentils, pear, artichoke, carrots, oatmeal, broccoli, and apples are examples of diet rich in fiber [37]

Fish oil	(i) Fish oil rich in omega-3 fatty acids may inhibit the promotion and progression of cancer through suppression of arachidonic acid-derived eicosanoid biosynthesis, which results in altered immune response to cancer and modulation of inflammation, cell proliferation, apoptosis, metastasis, and angiogenesis [38] (ii) It also influences transcription factor activity, gene expression, and signal transduction, which leads to changes in metabolism, cell growth, and differentiation [38–40]

Olive oil	(i) Olive oil reduces deoxycholic acid in the human colon and rectum (ii) Deoxycholic acid was found to reduce diamine oxidase, a main enzyme for the metabolism of ingested histamine and control of mucosal proliferation in the ileal and the colonic mucosa [41]

Folate	(i) Folate acts as donors of methyl groups in the biosynthesis of nucleotide precursors used for DNA synthesis and methylation of DNA, RNA, and protein and participates in the maintenance of genomic stability [42, 43] (ii) Spinach, broccoli, strawberries, raspberries, beans, peas, lettuce, lentils, and celery are examples of diet rich in folate [37]

Calcium	(i) Calcium can suppress epithelial cell proliferation in the colon by binding to bile acids and ionized fatty acids [44] (ii) Calcium can act directly by reducing proliferation, stimulating differentiation, and inducing apoptosis via upregulation of p21 and Bcl-2 in the colonic mucosa [44–47]

**Table 6:** Methods of allelic discrimination used in SNP genotyping [63].

Enzymatic cleavage	Enzymatic cleavage is based on the ability of certain classes of enzymes to cleave DNA by recognition of specific sequences and structures. Such enzymes can be used for discrimination between alleles when SNP sites are located in an enzyme recognition sequence and allelic differences affect recognition. For example, restriction fragment length polymorphism (RFLP) is based on genotyping a SNP located in a restriction enzyme site using PCR product containing the SNP that is incubated with corresponding restriction enzyme. The reaction product is run on a gel, and SNP genotype is easily determined from the product sizes [64].

Primer extension	In a typical primer extension reaction, a primer is designed to anneal with its 3^\^ end adjacent to a SNP site and extended with nucleotides by polymerase enzyme. The identity of the extended base is determined either by fluorescence or mass to reveal SNP genotype, for example, the PinPoint assay, MassEXTEND tm, SPC-SBE, and GOODassay primer extension-based methods, where SNP-specific primers are simultaneously extended with various nucleotides using PCR products as a template [65].

Hybridization	Hybridization approaches use differences in the thermal stability of double-stranded DNA to distinguish between perfectly matched and mismatched target-probe. For example, the TaqMan® genotyping assay combines hybridization and 5^\^ nuclease activity of polymerase coupled with fluorescence detection. The allele-specific probes carry a fluorescent dye at one end (reporter) and a nonfluorescent dye at the other end (quencher). The intact probes show no fluorescence owing to the close proximity between the reporter and quencher dyes. During PCR primer extension, the enzyme only cleaves the hybridized probe that is perfectly matched, freeing the reporter dye from the quencher. The reporter dye generates a fluorescent signal, whereas the mismatched probe remains intact and shows no fluorescence [66].

Ligation	Ligation approach employs specificity of ligase enzymes. When two oligonucleotides hybridize to single-stranded template DNA with perfect complementarity, adjacent to each other, ligase enzymes join them to form a single oligonucleotide. Three oligonucleotide probes are used in traditional ligation assays, 2 of which are allele-specific and bind to the template at the SNP site. The third probe is common and binds to the template adjacent to the SNP immediately next to the allele-specific probe. For example, combinatorial fluorescence energy transfer tags are composed of fluorescent dyes that can transfer energy when they are in close proximity. Tags with different fluorescence signatures can be created using a limited number of dyes by varying the number of dyes used and spacing between the dyes [67].

**Table 7:** Some of the published GWASs on CRC (100).

Reference SNP (rs)	Gene or region	Population	Sample size for stage	Sample size for subsequent stages	Genotyping platform (Nb. of SNPs)	Study reference
rs4939827	18q21 SMAD7	First stage: UK	940 cases/965 controls	7473 cases/5984 controls	Affymetrix (550,163)	(101)
		Second stage: UK				
rs6983267	8q24	First stage: UK	930 cases/960 controls	7334 cases/5246 controls	Illumina (547,647)	(102)
		Second stage: UK				
rs10505477	8q24	First stage: Canada	1257 cases/1336 controls	4024 cases/4042 controls	Illumina and Affymetrix (99,632)	(103)
rs719725	9p24	Other stages: Canada, US, and Scotland				
rs4779584	15q13 CRAC1	First stage: UK	730 cases/960 controls	4500 cases/3860 controls	Illumina (547,647)	(104)
		Second stage: UK				
rs4939827	18q21 SMAD7	First stage: Scotland	98 cases/1002 controls	16476 cases/15351 controls	Illumina (541,628)	(105)
rs7014346	8q24	Second stage and replication: Canada, UK, Israel, Japan, and EU				
rs3802842	11q23					
rs4444235	14q22.2 BMP4	First stage: UK	6780 cases/6843 controls	13406 cases/14012 controls	Multiple (38,710)	(106)
rs9929218	16q22.1 CDH1	Replication: EU, Canada				
rs10411210	19q13 RHPN2					
rs961253	20p12.3					

**Table 8:** Gene polymorphisms associated with CRC.

Gene	Reference SNP (rs)	Effect on CRC	Reference
Matrix metalloproteinases-9 (MMP 9)	rs34016235	A promoter polymorphism due to a C to T substitution results in the loss of the binding site of a nuclear protein to this region of the MMP 9 gene promoter. The polymorphism is associated with lymph node metastasis of CRC.	[78]
COX-2	rs20417	The C allele has lower promoter activity than the G allele, and GG genotype in smokers is associated with a significant increase in the risk of CRC compared to nonsmokers.	[79]
Vitamin D receptor	rs1544410	Polymorphism of the vitamin D receptor gene to be associated with an increased risk of colon cancer.	[80]
Bone morphogenetic protein 4 (BMP 4)	rs4444235	The rs4444235 increases risk of CRC development through its cis-acting regulatory influence on BMP4 expression.	[81]
Phospholipase A2	rs9657930	Polymorphisms in the phospholipase A2 gene is associated with the risk of the rectal cancer.	[82]
Colorectal adenoma and carcinoma 1	rs4779584	The rs4779584 polymorphism is associated with increased risk of CRC among Caucasian not Asian populations.	[83]
Eukaryotic translation initiation factor 3	rs16892766	The rs16892766 polymorphism is associated with increased risk of CRC but not adenoma among Caucasian.	[84]
Cadherin-1	rs9929218	The minor allele of rs9929218 has reduced E-cadherin expression and resulted in worsening the survival of CRC patients.	[85]
FAS	rs2234767	The rs2234767 contributes to an increased risk of CRC by altering recruitment of SP1/STAT1 complex to the FAS promoter for transcriptional activation.	[86]
Maternally expressed gene 3	rs7158663	The rs7158663 changes the folding structures of maternally expressed gene 3; therefore, it contributes to genetic susceptibility of CRC.	[87]
Fc-g receptor gene	rs1801274	The rs1801274 changes the amino acid from histidine (H) to arginine. CRC patients with Fc-g receptor H/H genotype have better survival.	[88]
SPSB2 gene	rs11064437	The rs11064437 contributes to an increased risk of CRC by disrupting the splicing and introduction of a transcriptional isoform with a shortened untranslated region of SPSB2 gene.	[89]
TPP1	rs149418249	Prevents TPP1-TIN2 interaction, shortening the telomere length, and as a consequence, enhances cell proliferation	[90]
SLC22A5	rs27437	The G allele decreases the expression of SLC22A5 via influencing the TF-binding upstream of the gene, leading to higher CRC risk.	[91]
*KBTBD11*	rs11777210	C allele allows binding of MYC, a potent oncogene, preventing the expression of KBTBD11, a potent tumor suppressor.	[92]
miR-17-92 cluster	rs9588884	The G allele lowers the CRC risk by decreasing transcriptional activity and consequently lowering levels of miR-20a.	[93]

**Table 9:** The role of TGF-*β* in various cell processes.

Cytostasis	(i) TGF-*β* can activate cytostatic gene responses at any point in the cell cycle phases G1, S, or G2 [112] (ii) TGF-*β* induces activation of the cyclin-dependent kinase (CDK) inhibitors [113–115] and repression of the growth-promoting transcription factors c-MYC and inhibitors of differentiation (ID1, ID2, and ID3) [116].

Apoptosis	TGF-*β* induces apoptosis through (i) upregulation of SH2-domain-containing inositol-5-phosphatase expression, which inhibits signaling via the survival protein kinase AKT [117] (ii) induction of TGF-*β*-inducible early-response gene, which induces the generation of ROS and the loss of the mitochondrial membrane potential preceding the apoptotic death [118, 119] (iii) induction of death-associated protein kinase [117]

Immunity	For immune suppression, TGF-*β* plays a critical role through (i) blocking antigen-presenting cells such as dendritic cells, which acquire the ability to effectively stimulate T cells during an immune response [120] (ii) decreasing the activity of natural killer cells and neutrophils [121]

Angiogenesis	(i) TGF-*β* induces the expression of matrix metalloproteinases (MMPs) on both endothelial cells and tumor cells, allowing the release of the endothelial cells from the basement membrane [122] (ii) TGF-*β* can also induce the expression of angiogenic factors such as vascular endothelial growth factor (VEGF) and connective-tissue growth factor (CTGF) in epithelial cells and fibroblasts [123, 124]

Epithelial-mesenchymal transition (EMT)	The migratory ability of epithelial cells relies on loss of cell–cell contacts, a process that is commonly referred to as the EMT. It is marked by the loss of E-cadherin and the expression of mesenchymal proteins such as vimentin and N-cadherin [125]. (i) TGF-*β* was reported to destabilize the E-cadherin adhesion complex resulting in its loss in pancreatic cancer [126]. Alternatively, in epithelial cell lines, TGF-*β* can deacetylate the E-cadherin promoter, thus repressing its transcription [127] (ii) TGF-*β* was found to upregulate vimentin in prostate cancer [128] (iii) TGF-*β* upregulates MMPs to promote invasion through proteolytic degradation and remodeling of the ECM [129]

**Table 10:** TGF-*β*-induced non-Smad signaling pathways.

c-Jun N-terminal kinases (JNK)/p38 activation	(i) TGF-*β* can rapidly activate JNK and p38 through MAPK kinases (MKK4, MKK 3/6) in various cell lines [133, 134]. Activation of JNK/P38 plays a role in TGF-*β*-induced apoptosis and in TGF-*β*-induced EMT [135].
Extracellular signal-regulatedkinase (ERK) activation	(i) TGF-*β* was found to activate the mitogen-activated protein kinase (MAPK)/extracellular signal-regulated kinase (ERK) pathway which are important for TGF-*β* mediated EMT [125, 136].
Phosphoinositide 3-kinase(PI3-K)/AKT activation	(i) TGF-*β* was reported to rapidly activate phosphoinositide 3-kinase (PI3-K) as indicated by the phosphorylation of its downstream effector Akt [137] (ii) Although the PI3-K/Akt pathway is a non-Smad pathway contributing to TGF-*β*-induced EMT, it can antagonize Smad-induced apoptosis and growth inhibition [138]
Rho-like GTPases	(i) The Rho-like GTPases, such as Ras homolog gene family, member A (RhoA) plays an important role in controlling dynamic cytoskeletal organization, cell motility, and gene expression and is a key player in TGF-*β*-induced EMT [139] (ii) TGF-*β* regulates RhoA activity in two different modes as it induces a rapid activation of RhoA during the early phase of stimulation and then downregulates the level of RhoA protein at later stages, both of these modes of regulation appear to be essential for TGF-*β*-induced EMT [140]

**Table 11:** Alterations of some components of TGF-*β* pathway in human tumors.

TGF-*β*R2	(i) The TGF-*β*R2 gene has been mapped to chromosome 3p, a chromosome in which mutation was observed in small cell lung carcinoma (SCLC), non-small-cell lung carcinoma (NSCLC), CRCs, and ovarian and breast cancers [142–144] (ii) Besides mutations in the coding region of TGF-*β*R2, loss of expression of TGF-*β*R2 in NSCLCs, bladder cancer, and breast cancer were reported [145–147]

TGF-*β*R1	(i) The TGF-*β*R1 gene has been mapped to chromosome 9q (ii) Mutation in TGF-*β* gene was reported in ovarian cancer, head and neck squamous cell carcinomas (HNSCC), and breast cancer [148–150] (iii) Homozygous deletion of TGF-*β*R1 was also identified in pancreatic and biliary adenocarcinomas [151]

SMAD3	(i) The gene for SMAD3 is located in chromosome 15q21-q22 (ii) The rate of mutation in the SMAD3 gene is rare, and there are only few examples of such defects in Smad3 expression that was found in some gastric cancer and leukemia [152, 153]

SMAD2/SMAD4 and SMAD7	(i) Chromosome 18q has genes encodes for SMAD2, SMAD4, and SMAD7 (ii) Mutation in chromosome 18q was found in about 30% of neuroblastoma, breast, prostate, and cervical cancers and even more frequently in HNSCC (40%), NSCLC (56%), colon cancer (60%), gastric cancer (61%), and 90% of pancreatic tumors [154, 155]

**Table 12:** Association studies of SNPs in SMAD7 gene and CRC.

Population	Reference SNP (rs)	Location	Association	Reference
African American and Caucasian	rs4939827	Intron 3	In women: yes	[166]
	rs4464148	Intron 3	Yes	
Caucasian	rs12953717	Intron 3	Yes	[167]
	rs4939827	Intron 3	Yes	
	rs4464148	Intron 3	No	
Swedish	rs4939827	Intron 3	Yes	[168]
European	rs4464148	Intron 3	Yes	[169]
	rs4939827	Intron 3	No	
Chinese	rs4939827	Intron 3	No	[170]
	rs12953717	Intron 3	Yes	
	rs4464148	Intron 3	No	
African American	rs4939827	Intron 3	Yes	[171]
Chinese	rs4939827	Intron 3	Yes	[76]
Romanian	rs4939827	Intron 3	CRC vs control: no	[172]
			Rectal vs colon cancer: yes	
Caucasian	rs4939827	Intron 3	Yes	[173]
Croatian	rs4939827	Intron 3	Yes	[77]
Italian	rs4939827	Intron 3	Yes	[174]
Korean	rs4939827	Intron 3	Yes	[175]
Spanish	rs4939827	Intron 3	Yes	[176]
French	rs4939827	Intron 3	Yes	[177]
	rs58920878	Intron 3	Yes	

**Table 13:** Serum YKL-40 levels (ng/ml) in patients with inflammation, tissue remodeling, or fibrosis [201].

Disease	Median serum YKL-40 (ng/l)	Reference
Viral hepatitis	83	[202]
Noncirrhotic fibrosis	158	
Posthepatitis cirrhosis	204	
Rheumatoid arthritis	110	[203]
Streptococcus pneumoniae bacteremia	342	[200]
Osteoarthritis	112	[204]
UC, severe	59	[205]
CD, severe	59	
Pulmonary sarcoidosis	201	[206]
Asthma	92	[207]

**Table 14:** Serum YKL-40 levels (ng/ml) in patients with localized or advanced cancer [201].

Disease	Median serum YKL-40 (ng/l)	Reference
Metastatic breast cancer	80	[209]
CRC	160	[210]
Glioblastoma multiforme	130	[195]
Lower grade gliomas	101	
Primary breast cancer	57	[211]
Small cell lung cancer	82	[192]
Local disease	71	
Extensive disease	101	
Metastatic prostate cancer	112	[208]
Ovarian cancer, all stages	94	[212]
Ovarian cancer, stage III	168	
Ovarian cancer, relapse	94	

**Table 15:** Association of some CHI3L1 SNPs with diseases.

Disease	Population	Reference SNP (rs)	Location	Association	Reference
Sarcoidosis	Caucasian	rs10399931	Promoter	No	[226]
Schizophrenia	Caucasian	rs10399805	Promoter	Yes	[163]
Liver fibrosis	Caucasian	rs4950928	Promoter	Yes	[225]
Glioblastoma	Caucasian	rs4950928	Promoter	No	[227]
Asthma and atopy	Danish	rs4950928	Promoter	Yes	[228]
Rheumatoid arthritis	Danish	rs4950928	Promoter	No	[229]
		rs6691378	Promoter	No	
		rs10399931	Promoter	No	
		rs880633	Exon 5	No	
Coronary heart disease	Chinese	rs10399931	Promoter	No	[230]
Schizophrenia	Japanese	rs4950928	Promoter	Yes	[231]
Atrial fibrillation	Danish	rs4950928	Promoter	No	[232]
Asthma	African Americans	rs4950928	Promoter	Yes	[233]
Cervical cancer	Taiwanese	rs10399805	Promoter	Yes	[234]
		rs4950928	Promoter	No	
Asthma	Taiwanese	rs10399931	Promoter	Yes	[235]
		rs1538372	Intron2/exon3	Yes	
Atherosclerosis	Taiwanese	rs10399931	Promoter	No	[236]
Asthma	Indian	rs4950928	Promoter	No	[237]
Non-Hodgkin's lymphoma	Danish	rs4950928	Promoter	Yes	[238]
Asthma	Swedish	rs4950928	Promoter	No	[239]
Venous thromboembolism	Danish	rs4950928	Promoter	No	[240]
Coronary artery disease	Taiwanese	rs4950928	Promoter	Yes	[241]
